# Endocan and Circulating Progenitor Cells in Women with Systemic Sclerosis: Association with Inflammation and Pulmonary Hypertension

**DOI:** 10.3390/biomedicines9050533

**Published:** 2021-05-11

**Authors:** Alberto Lo Gullo, Giuseppe Mandraffino, Javier Rodríguez-Carrio, Michele Scuruchi, Davide Sinicropi, Maria Postorino, Carmela Morace, Clemente Giuffrida, Davide Sciortino, Romina Gallizzi, Saverio Loddo, Concetta Zito, Giovanni Squadrito

**Affiliations:** 1 Medicine and Urgency Unit, Piemonte Hospital, IRCCS Neurolesi Bonino Pulejo, 98121 Messina, Italy; clemente.giuffrida@irccsme.it; 2 Internal Medicine Unit, Department of Clinical and Experimental Medicine, University of Messina, 98125 Messina, Italy; sinicropidavide@gmail.com (D.S.); mariapostorino@hotmail.it (M.P.); cmorace@unime.it (C.M.); gsquadrito@unime.it (G.S.); 3 Area of Immunology, Department of Functional Biology, Faculty of Medicine, University of Oviedo, 33006 Oviedo, Spain; rodriguezcjavier@uniovi.es; 4 Instituto de Investigación Sanitaria Del Principado de Asturias (ISPA), 33011 Oviedo, Spain; 5 Bone and Mineral Research Unit, Instituto Reina Sofía de Investigación Nefrológica, REDinREN Del ISCIII, Hospital Universitario Central de Asturias, 33011 Oviedo, Spain; 6 Molecular Biology Lab, Department of Clinical and Experimental Medicine, University of Messina, 98125 Messina, Italy; mscuruchi@unime.it; 7 Rheumatology Unit, Department of Clinical and Experimental Medicine, University of Messina, 98125 Messina, Italy; davsciortino@gmail.com; 8 Unit of Pediatrics, Department of Human Pathology in Adulthood and Childhood, University of Messina, 98125 Messina, Italy; rgallizzi@unicz.it; 9 Pediatric Unit, Department of Medical of Health Sciences, Magna Graecia University, 88100 Catanzaro, Italy; 10 Laboratory Medicine, Department of Clinical and Experimental Medicine, University of Messina, 98125 Messina, Italy; sloddo@unime.it; 11 Cardiology Unit, Department of Clinical and Experimental Medicine, University of Messina, 98125 Messina, Italy; czito@unime.it

**Keywords:** endocan, circulating progenitor cells, pulmonary hypertension, systemic sclerosis

## Abstract

Background: Systemic sclerosis (SSc) is characterized by early vasculopathy and fibrosis in the skin, lungs, and other tissues. Vascular manifestations of SSc include Raynaud’s phenomenon, digital ulcers, and pulmonary artery hypertension (PAH). PAH is the second most common cause of mortality in SSc. Circulating CD34+ cells associated with cardiovascular health status in several conditions, including chronic immune-inflammatory disease. CD34+ cell numbers have been found inconstantly reduced in SSc. Endocan, a proteoglycan expressed by endothelial cells, was recently suggested as a marker of vascular stress. We tested the relationships among CD34+ cells, endocan, inflammatory markers, vitamin D levels, and clinical parameters in SSc patients with PAH. METHODS: Standard echocardiography was performed. Vitamin D levels, CD34+ cells, inflammatory markers, endocan plasma levels were determined in 36 female SSc patients (24 diffuse/12 limited) and 36 matched controls (HC). RESULTS: We found no difference in CD34+ and vitamin D levels in SSc as compared to controls; ESR, CRP, fibrinogen, endocan, sPAP were higher in SSc with respect to controls. We found a correlation between endocan and: CD34+ cells (r: −0.540, *p* = 0.002), pulmonary arterial pressure (sPAP) (r: 0.565, *p* < 0.001), tricuspid annular plane excursion (TAPSE) (r: −0.311, *p* < 0.01), and E/A ratio (r: −0.487, *p* < 0.001), but not with ejection fraction (r: −0.057, *p* = 0.785) in SSc. CD34+ cells correlate with fibrinogen (r: −0.619, *p* < 0.001), sPAP (r: −0.404, *p* = 0.011), E/A (r: 0.470, *p* < 0.005 in SSc. CONCLUSION: CD34+ cell number was significantly correlated with endocan levels and with sPAP in SSc; endocan and CD34+ progenitor cells might be suggested as a potential marker of disease status.

## 1. Introduction

Systemic sclerosis (SSc) is an autoimmune disease characterized by skin and internal organ fibrosis, as well as vascular abnormalities [1,2], including endothelial dysfunction and pulmonary arterial hypertension (PAH) [2,3]. Endothelial dysfunction and vascular alterations in SSc progressively lead to hemodynamic changes and increased pulmonary arterial pressure in 7.85–13% of SSc patients [4], being one of the most frequent causes of death [5,6]. Despite being largely explored, prognostic biomarkers and predictive models to identify patients at risk of PAH are lacking [3,7].

Circulating proangiogenic hematopoietic cells (PHCs) are bone marrow-derived, multipotent circulating cells with the ability to differentiate into different cell types, such as cardiomyocytes, smooth muscle cells, endothelial progenitor cells (EPCs), and endothelial cells (ECs), thus participating in the turnover and remodeling vascular beds [8,9], including those in the lungs [10].

Several studies have shown that EPCs could be impaired in number or function in a broad range of immune-mediated diseases [11,12,13,14,15,16,17,18,19,20]. However, their role in SSc is still a matter of debate since conflicting reports have been released. Some studies have reported decreased CD34^+^/CD133^+^/VEGFR2^+^ cell counts in SSc by flow cytometry [14,15,21], whereas increased circulating EPC levels have been reported elsewhere [13,22,23]. Prolonged and continuous endothelial cell recruitment has been suggested to exhaust the bone marrow reservoir of resident EPCs, and the disease-related toxic mechanisms can negatively influence the half-life and mobilization of EPCs. Furthermore, immune dysregulation has been described within the bone marrow environment in SSc [11,24]. Therefore, bone marrow and stromal cell dysfunction may lead to an altered progenitor cell reserve and mobilization capacity, hence hampering their homeostatic functionality and leading to PAH at the lung level [25].

A number of soluble mediators are known to modulate the bone marrow-vascular axis in endothelial dysfunction, including vitamin D and several inflammatory markers. Decreased vitamin D levels have been related to adverse CV events [26] and inflammation-linked vascular endothelial dysfunction [27]. Furthermore, vitamin D might have a role in the proliferation and differentiation of stem cells toward endothelial phenotype by modulating phospholipase C, VEGF1 and 2, and pro-MMP-2 activity [28,29,30]. Therefore, vitamin D is thought to be key to maintain endothelium homeostasis in chronic conditions [31]. On the other hand, endothelial cell-specific molecule-1 (ESM-1, or endocan) is a soluble dermatan sulfate proteoglycan, secreted preferentially by vascular endothelial cells in response to different stimuli such as tumor necrosis factor α (TNFα) and the proangiogenic factor vascular endothelial growth factor (VEGF) [32], among others. Endocan participates in the molecular interactions underlying several biological processes, including cell adhesion, migration, proliferation as well neo-intima formation during vascular traits [32,33,34,35,36,37]. Endothelial activation and inflammation increase the endocan expression and release, which in turn fuels vascular inflammation. Therefore, endocan has been proposed as a biomarker of endothelial dysfunction and pathological angiogenesis [32,34], thus suggesting its usefulness as a potential predictor of a wide spectrum of CV outcomes [38,39,40] and pulmonary disease [41,42].

Taken together, it may be conceived that PHCs [43] and endocan could be involved in the development of PAH, but their interrelationships have not been investigated so far in SSc. Therefore, we designed an observational cross-sectional study to investigate the relationships between CD34+ PHCs, endocan, inflammatory markers, vitamin D levels, and clinical parameters in SSc patients with PAH.

## 2. Materials and Methods

**Figure 1:**
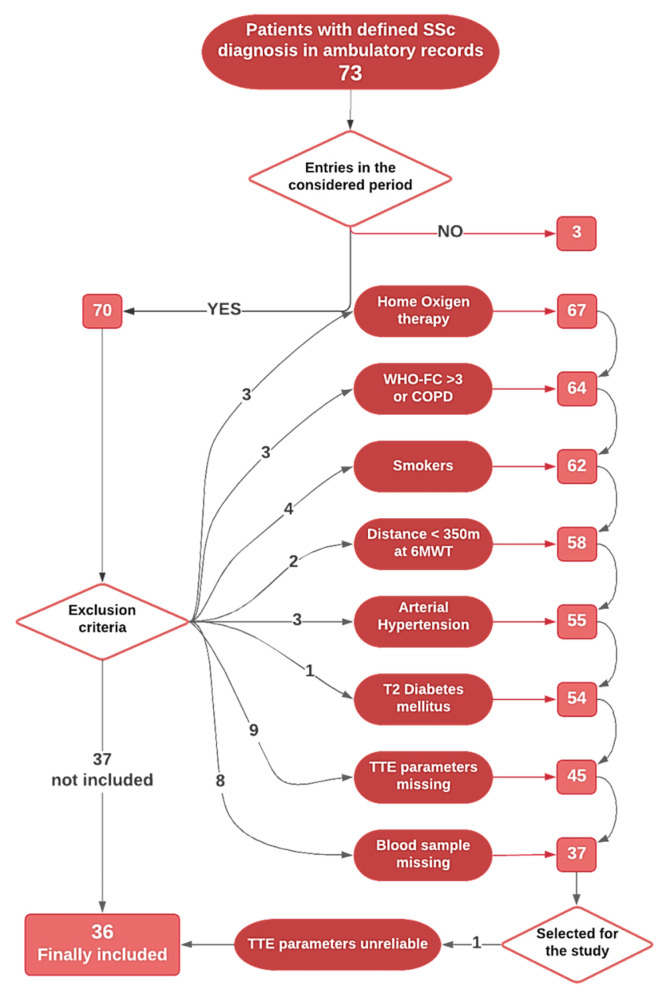
Flow diagram for inclusion/exclusion path. 73 records found regarding female patients with diagnosis confirmed of SSc; 3 patients did not have entries relating to the period considered; 3 had started O2 therapy; 9 had COPD, or were assigned to WHO FC > 3, or were smokers, or walked a distance < 350 m; 4 subjects were excluded due to relevant comorbidities (3 for arterial hypertension, 1 for type 2 diabetes mellitus); 9 were not eligible due to missing needed echocardiography parameters; 8 were excluded because we did not find the blood sample stored in our bio-bank; finally, 1 patient was excluded after the primary selection because of the unreliability of echocardiography parameters.

Overall, all the patients recorded with a definite diagnosis of SSc to the Unit of Rheumatology of our Department of Clinical and Experimental Medicine, University of Messina, were checked for eligibility, finding 81 patients. Eight male patients were immediately excluded; starting with 73 female patients, thirty-six SSc female inpatients hospitalized from December 2010 to March 2016 were finally recruited. The inclusion/exclusion path is represented in Figure 1. Patients with respiratory functional impairment (oxygen therapy, chronic obstructive pulmonary disease or COPD, WHO-FC > 3, 6-min walking distance < 350 m, DLCO < 60%) or smokers were excluded, as well patients with relevant comorbidities (arterial hypertension, type 2 diabetes mellitus). In order to avoid seasonal variation of vitamin D levels, recruitment and blood sampling were scheduled between October and May only. The limited and diffuse SSc disease subsets were classified according to EULAR criteria [44]. Disease duration was defined as the first non-Raynaud’s symptom [45]. According to 2015 ESC/ERS recommendations, a value of sPAP > 36 mmHg was defined as “possible pulmonary hypertension” [46]. Rodnan skin score (RSS) was registered [45,47]. As the control group, thirty-six healthy subjects to be age- and sex-matched were selected from our database (collecting the data of the hospital personnel who provided the consent to serve as a control in observational studies). All participants were Caucasians from Southern Italy. The Local Ethics Committee for Medical Research at the University Hospital of Messina approved the study (Prot. N. 11/17), and it was carried out in accordance with the Helsinki Declaration. All subjects provided their informed written consent in order to be enrolled.

### 2.1. Clinical Parameters and Biochemical Data

Blood samples were obtained by antecubital venipuncture between 8 and 9 a.m. after an overnight fast and a 10-min rest. Each patient underwent a complete clinical evaluation and clinical chemistry, including complete blood count with formula, hemoglobin levels, and inflammation indices (C-reactive protein—CRP; erythrocyte sedimentation rate—ESR; fibrinogen), serum uric acid levels (SUA), serum creatinine.

Echocardiography study, including 2-dimensional study in the parasternal axis, apical and subcostal views, was performed in double by two experienced sonographers using a GE Vivid 7 or a GE Vivid E9 (GE Vingmed Ultrasound AS, Horten, Norway) cardiac ultrasound machine. All cardiac parameters, including systolic and diastolic function of the left and right ventricles, were assessed following current recommendations of the American Society of Echocardiography (ASE) [48]. In detail, sPAP was calculated as the sum of the estimated right atrial pressure (eRAP) by inferior vena cava diameter, and the peak velocity of the tricuspid regurgitant jet (TRV), as following: sPAP = 4*TRV^2^ + eRAP. For standardization, an eRAP of 10 mmHg was assumed for all patients unless clear features were present that suggested otherwise. An apical RV-focused four-chamber view of the systolic longitudinal displacement of the lateral tricuspid annulus toward the apex was evaluated to measure TAPSE by using M-mode echocardiography. Ejection fraction (EF) was calculated according to the Simpson method. Analyzing the right heart was performed by assessing the right atrium (RA) size, right ventricle (RV) size, and RV wall thickness. In pulsed-wave Doppler imaging, LV diastolic parameters were studied: peak velocity of early (E) and late (A) transmitral filling of LV and their ratio (E/A), isovolumetric relaxation time (IVRT) of LV. RA indexed volume and the right ventricle outflow tract (RVOT) proximal diameter were also estimated as a measure of RV dimension [46,48].

The 6MWT was performed as per American Thoracic Society guidelines. All patients were tested in room air without additional oxygen under standardized conditions in the same pulmonary function laboratory by trained technicians not involved in the patients’ daily care. Patients were instructed as follows: “The object of this test is to walk as far as possible for 6 min. You will walk back and forth in this hallway. Six minutes is a long time to walk, so you will be exerting yourself. You will probably become out of breath or become exhausted. You are permitted to slow down, to stop, and to rest as necessary. You may lean against the wall while resting, but resume walking as soon as you are able.” Baseline blood pressure, heart rate, and saturation on pulse oximetry (SpO2) were measured. Special attention was paid to the SpO2 pulse signal. SpO2 and heart rate were monitored throughout the walk. Total distance walked (6-min walking distance, 6MWD) was recorded. The predicted six-minute walk distance (6MWD) for women was calculated based on the following formulas (2.11 × height) − (2.29 × weight) − (5.78 × age) + 667 m. The 6MWD was considered abnormal if it was <80% of the normal range predicted by Enright’s equation [49]. We considered a distance walked of at least 350 m as the inclusion criterion [50].

### 2.2. Laboratory Analyses

Vitamin D_3_ was measured using high-performance liquid chromatography (Bio-Rad, München, Germany); endocan plasma levels were measured by a commercially available ELISA kit (Cohesion Biosciences).

CD34+ cell numbers were obtained as previously detailed [51]. Briefly, flow cytometry (FACSCalibur; Becton Dickinson and Co., Franklin Lakes, NJ, USA) was used to identify and count circulating CD34+ cells from peripheral blood. Staining and analysis were performed using the International Society of Hematotherapy and Graft Engineering (ISHAGE) sequential gating strategy; a multiparameter flow cytometric lyse no-wash method PROCOUNT (BD) in a TRUCOUNT tube (BD) with a known number of fluorescent beads was used. Gating strategies and sample analyses allowed the identification of CD34+ cells by using the Macintosh CELLQuest software program (BD). This kind of acquisition, processing, and evaluation allows presenting the data as absolute cell count per microliter of peripheral blood [20].

### 2.3. Statistical Analysis

The Kolmogorov–Smirnov test was used to test the distribution of the variables. Despite the low sample size, most of the studied variables presented with a normal distribution (endocan, CD34+ cells, vitamin D levels, fibrinogen). Consistently, a standard parametric approach was used. Variables were summarized as mean ± SD. Groups were compared using the Student *t*-test. The correlations among the variables were assessed by the Pearson test. To assess the contribution of each variable on study variables, a linear, stepwise, multivariate regression analysis was performed. A two-tailed alpha of 0.05 was used to indicate statistical significance. IBM SPSS statistics for Mac (ver. 26, IBM Corporation) was used to analyze the statistical data.

## 3. Results

As reported, Figure 1 shows the inclusion/exclusion path; briefly, Table 1 summarizes the main demographic data of the participants. Thirty-six female SSc patients were included, mean age 64.1 ± 12.5 years, and compared to 36 healthy controls (62.3 ± 4.4 years).

The clinical characteristics of SSc patients were summarized in Table 2; we recorded disease duration, the pattern of SSc (diffuse/limited), the presence of anticentromere antibodies (ACA), anti-topoisomerase I (Anti-Scl70), antinuclear autoantibodies (ANA); pulmonary and musculoskeletal involvement as well as previous renal crisis and esophageal reflux were also evaluated.

Fibrinogen, CRP, and ESR values were higher in SSc patients compared to controls. Twelve SSc patients presented with vitamin D levels < 20 ng/mL, nine patients with vitamin D levels between 20 and 30 ng/mL, and 16 patients with normal levels.

We found endocan levels significantly higher in SSc patients as compared to controls: 365.6 ± 225.9 vs. 280.4 ± 68.7, *p* = 0.03; vitamin D levels were 27.34 ± 10.8 vs. 22.47 ± 2.6, *p* = 0.010. CD34+ cell number did not differ between SSc and controls: 2.68 ± 0.79 vs. 2.61 ± 0.17, *p* = 0.604.

We performed a separate statistical analysis in controls and in patients.

We found no difference as regards endocan, vitamin D levels, and CD34+ cell number in patients with diffuse subtype SSc as compared with patients with limited SSc. NO association between BMI and endothelial markers nor vitamin D in SSs.

Since we found a significant difference between SSc patients and controls as regards the main study variables, we performed the statistical analyses in the two groups separately. Moreover, we tried to evaluate whether diffuse and limited forms of SSc presented with different interrelationships.

**Figure 2:**
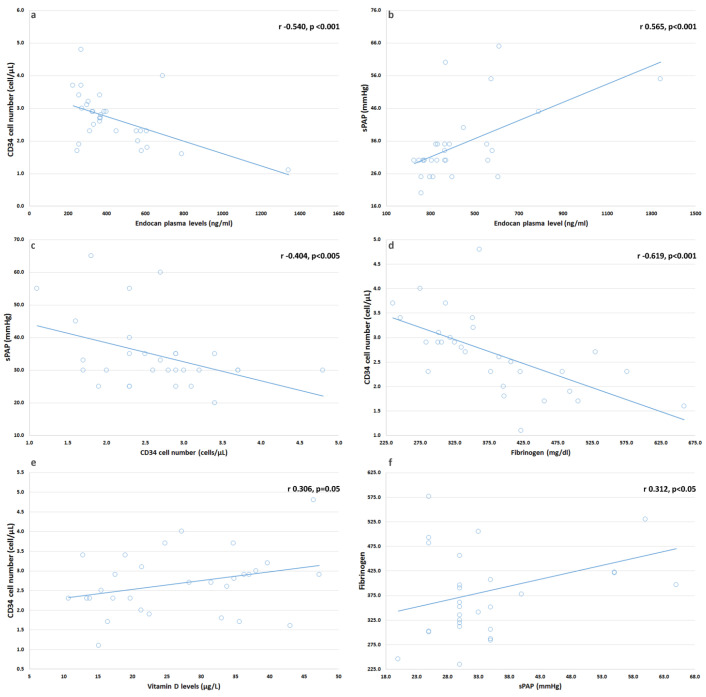
Spearman’s correlation between CD34+ and endocan (**a**); sPAP and endocan (**b**); sPAP and CD34+ (**c**); CD34+ and fibrinogen (**d**); CD34+ and Vitamin D (**e**); sPAP and fibrinogen (**f**).

In SSc, an inverse correlation between endocan and CD34+ cell number (r: −0.540, *p* = 0.002), and between endocan and sPAP (r: 0.565, *p* < 0.001) was found (Figure 2a,b, respectively); moreover, endocan levels also have a negative correlation with TAPSE (r −0.311, *p* < 0.01), and E/A ratio (r: −0.487, *p* < 0.001), but not with EF (r: −0.057, *p* = 0.785). CD34+ cell number inversely correlates with sPAP (r: −0.404, *p* = 0.011, Figure 2c) and fibrinogen (r: −0.619, *p* < 0.001, Figure 2d), and positively with E/A (r: 0.470, *p* < 0.005); we also found a correlation coefficient next to the significance level for vitamin D levels (r: 0.306, *p* = 0.05, Figure 2e), while ESR (r: −0.206, *p* = 0.25) seemed not to correlate with CD34+ cell number, vitamin D levels do not correlate with CRP (r: −0.109, *p* = 0.451). Furthermore, we found a significant correlation between fibrinogen and sPAP (r: 0.312, *p* < 0.05, Figure 2f).

We found no difference as regards endocan, vitamin D levels, and CD34+ cell number in patients with diffuse subtype SSc as compared with patients with limited SSc. No association between BMI and endothelial markers nor vitamin D in SSc.

We found no significant correlation among control subjects as regards the main study variable (endocan, sPAP, vitamin D, fibrinogen, CRP, CD34+ cells).

We also tested the dependence of: CD34+ cells number, CRP, fibrinogen, sPAP, and also endocan on duration disease (in months); we found a significanst value only as regards CRP values (F 5.533, *p* = 0.025).

In order to evaluate the contribution of each variable on CD34+ cell number, we carried out a linear regression analysis among SSc patients only (we tested the dependence from endocan, vitamin D levels, CRP, fibrinogen, SUA, sPAP); dependence analysis suggested that endocan (β = −0.514; *p* < 0.005), fibrinogen levels (β = −0.667; *p* < 0.001) and sPAP (β = −0.498; *p* = 0.006) are negatively associated with CD34+ cell number. These variables were selected for the multivariable regression analysis; endocan (β = −0.412, *p* = 0.006) and fibrinogen (β = −0.386, *p* = 0.009) were indicated as potential predictors of CD34+ cell number (Table 3 and Table 4).

Table 3 and Table 4 shows the regression analysis performed to verify the dependence of CD34+ cell number on the study variables; Table 3: linear regression analysis; Table 4: multivariable regression analysis; B: unstandardized regression coefficient; SE: standard error for B; Beta: standardized regression coefficient; t: non-parametric *t*-test for beta; *p*: *p*-value for significance.

In a second regression model, we tested the dependence of sPAP from fibrinogen, CD34+ cell number, endocan, Rodnan skin score and SUA, finding that the variable associated to sPAP were endocan (β = 0.607; *p* < 0.001), fibrinogen (β = 0.471; *p* = 0.003), CD34+ cell number (β = −0.424; *p* = 0.009).

These variables were selected for the multivariable regression analysis; endocan only (β = 0.607, *p* < 0.001) was indicated as potential predictor of sPAP (Table 5 and Table 6).

Table 5 and Table 6 shows the regression analysis performed to verify the dependence of sPAP on the study variables; Table 5: linear regression analysis; Table 6: multivariable regression analysis; B: unstandardized regression coefficient; SE: standard error for B; Beta: standardized regression coefficient; t: non-parametric *t*-test for beta; *p*: *p*-value for significance.

The results showed that sPAP is influenced mainly by endocan levels but also seems to be affected by inflammatory status and by CD34+ levels.

### Vitamin D Supplementation and Medical Therapy

According to the 2011 cornerstone in vitamin D levels classification, ref [52] vitamin D deficiency was defined as plasma levels of 25(OH)D3 < 20 ng/mL, insufficiency between 20 and 30 ng/mL, and normal > 30 ng/mL.

Consistently, vitamin D supplementation has been suggested in outpatients when vitamin D levels were below 20 ng/mL at diagnosis, as a standardized first-level prescription with 25,000 oral cholecalciferol monthly. At enrolment blood sampling, all selected SSc patients were already being supplemented with standard vitamin D treatment.

All the selected patients were also prescribed low-dose aspirin (100 mg per day) and nifedipine 20 mg per day (sustained-release tablets). Moreover, three patients were prescribed bosentan and four patients sildenafil. No patients were assuming steroids as background therapy. Patients also assuming other drugs for comorbidities were not included in the study.

Unfortunately, we have not been able to take into account the potential effect of these therapies.

## 4. Discussion

Pulmonary arterial hypertension represents a major comorbidity in SSc. PAH is a progressive condition characterized by pronounced hemodynamic derangement, increased pulmonary artery pressure, and resistance. Despite an improved understanding of the mechanisms and mediators underlying PAH, mediators with predictive value, especially beyond the clinical features, remain to be investigated.

### 4.1. In SSc Patients, We Found Serum Endocan Levels Increased and Related with sPAP

Elevated endocan levels have already been reported to be related to several diseases, including autoimmune diseases [53,54,55], CV disorders, and related complications [32,33,37,38,40,53,55]. High endocan levels were detected in autoimmune diseases as psoriasis [54], Behçet’s disease [55], and systemic lupus erythematosus [39], and were associated with inflammatory response and autoimmune disease activity scores [39,54,55]. Endocan also appeared to differentiate SSc subgroups since its levels were higher in diffuse SSc with respect to limited forms [56], suggesting this mediator for a role as a disease marker. Endocan is a proteoglycan produced in a readily available form, whose chemical characteristics confer high flexibility and binding properties, resulting in a multifunctional molecule. Expressed in different types of cells and tissues, this proteoglycan is principally released from the vascular endothelium of the lungs and kidneys. Several pro-inflammatory molecules, including the cytokine TNF-α and the pro-angiogenic factor VEGF-A, are involved in endocan expression. Moreover, endocan is upregulated in activated endothelium and inflammatory conditions. Endocan upregulation elicits pro-inflammatory effects as endothelial activation and proliferation, cell adhesion molecule expression, leukocyte migration, and neo-angiogenesis [32,36]. Endocan, in turn, stimulates the expression of inflammatory cytokines and chemokines and activates pro-inflammatory signaling pathways. Due to the altered expression of endocan and bioactive molecules, these effects may underlie many pathological conditions, shared by chronic inflammation and vascular alterations, including PAH [57]. Studies have shown that mice overexpressing TNF-α had right ventricular hypertrophy and pulmonary hypertension and that TNF-α increased endocan in rat lung microvascular endothelial cells; conversely, endocan knockdown attenuated the damage induced by TNF-α in these cells [57]. These observations support the concept of a reciprocal dialogue between endocan and inflammatory molecules, including TNF-α [58]. In connective tissue disease presenting with PAH, we can speculate that disturbed mutual signaling involving endocan and different molecules may trigger a combination of events that result in altered angiogenesis and remodeling, fibrosis, and an increase in pulmonary arterial pressure and resistance. Thus, endocan could be considered as a marker of pulmonary arterial hypertension.

### 4.2. We Found No Changes in CD34+ Cell Number in Our Patients

PHCs are bone marrow-derived cells able to differentiate into several cell types, including EPCs and ECs. EPCs are currently considered as important contributors to vascular repair of injured arteries, replacing dysfunctional ECs and suppressing neo-intima formation. A reduced number of EPCs is associated with endothelial dysfunction and increased CV risk [17,18,19,20]; also, the EPC number has also been postulated as an independent predictor of CV risk [17,51,59]. A reduced number of EPCs was shown in patients with rheumatic disease, providing further evidence for endothelial dysfunction in such a population [17,18,19,20,60].

Some observations indicated that in SSc patients, EPCs were compromised in their function [13,14,15,16]. Other studies reported a significant depletion in the count of CD34^+^/CD133^+^/VEGFR2^+^ cells by using flow cytometry [14,15,21], whereas further results showed increased EPC levels in SSc [22,23,54]. These different findings may be due to the difficulties in correctly assessing patients with different scleroderma subtypes, disease duration, and disease activity.

It has also been suggested that prolonged and continuous endothelial cell recruitment may exhaust the bone marrow reservoir of resident EPCs and that disease-related toxic mechanisms may negatively influence the half-life and mobilization of EPCs. Furthermore, immuno-mediated mechanisms were described in the bone marrow environment, and significant titers of anti-endothelial cell antibodies were detected in the plasma of bone marrow from SSc patients [11,24,61].

### 4.3. We Found a Correlation between CD34+ Cells and sPAP

Decreased numbers of EPCs were reported to be associated with worse hemodynamic and abnormally elevated concentrations of inflammatory mediators [62]. Conversely, intra-pulmonary EPCs were found to be elevated in patients with end-stage lung disease and increased sPAP [63]. Evidence suggested that bone marrow progenitor cells are recruited during pulmonary vascular remodeling [10], and that defective EPC mobilization and recruitment may result in increased pulmonary hypertension [25]. Bone marrow stem cells provide different cell types as endothelial-like cells and smooth muscle-like cells that potentially contribute to vascular healing or injury, remodeling, and fibrosis in physiological and pathological conditions. Therefore, pulmonary arterial remodeling can be associated with chronic inflammatory events and recruitment of progenitor cell types, as circulating fibrocytes and mesenchymal progenitor cells, and EPCs, which may sustain proliferative changes or offered protective effects. In our patients, there are no changes in the number of EPCs; however, a significant correlation between cells and increased sPAP occurs, suggesting that cell recruitment is not sufficient to prevent vascular alterations, probably because EPCs are ineffective in their function. One limitation of our study is that we have not studied the functional status of EPCs. A number of studies have shown that EPCs exhibit different positive effects. For example, EPCs can act through an immune-dependent mechanism, potentially involving the stimulation of natural killer cells [64], or through secretion of paracrine factors [65]; some paracrine factors released by EPCs have been revealed as potent inhibitors of apoptosis [66]. EPCs may also mediate therapeutic benefits in treated patients with PAH; endothelial colony-forming cells from PAH patients treated with treprostinil showed a hyper-proliferative phenotype and enhanced angiogenic potential in a mouse model [67], suggesting that EPCs may not only be promising cells for the treatment of several diseases but may also promote the benefits of additional therapy.

### 4.4. We Found a Correlation between Vitamin D and CD34+ Suggesting That Multiple Factors Might Contribute to CD34+ Cells and Endothelium Homeostasis in SSc

Emerging evidence indicates that 1a,25-(OH)2 vitamin D3 (vitamin D), besides its role in calcium homeostasis and bone metabolism, regulates immune function and inflammation [18], also playing an important role in the cardiovascular system [68,69] and CVD [26,70,71]. Therefore, strategies to prevent diseases related to vitamin D status are of great interest.

CD34+ cell number could be negatively affected by low levels of vitamin D. In fact, vitamin D could contribute to the proliferation and differentiation of stem cells by interacting with phospholipase C, VEGF1, and 2 and pro-MMP2 activity [28,72]. In addition, it was also proposed that vitamin D protects progenitor cells against oxidative stress [30]; RA patients with higher vitamin D levels presented with higher CD34+ cell number [18], while reduced CD34+ cells were characterized by increased intracellular ROS levels and imbalanced antioxidant enzymes [20]. In addition, vitamin D supplementation can further stimulate EPC migration and differentiation [18,28].

Additionally, it increases endothelial progenitor adhesion by dampening the inflammatory signals of TNF-α in vitro [73] and could improve EPCs viability in vitro [74].

Growing evidence suggests that vitamin D may modulate KLF-10 levels, which in turn may stimulate the activity of EPCs [75]. Other CV effects have been accounted to vitamin D, including the improvement of nitric oxide metabolism, to attenuate the effect of advanced glycosylation end products (AGEs) and modulate the endothelial pathways NFkB-mediated [27,74]. These in vitro studies suggest a potential therapeutic role for vitamin D in enhancing the endothelial integrity under the circumstances involving vascular injury through the improvement of EPCs features. Although a number of epidemiological data indicate an association between vitamin D deficiency and CVD, suggesting a potential protective role, and experimental studies seem to support the anti-fibrotic and anti-hypertrophic role of vitamin D [76], the interventional studies with calcitriol or VDR agonists only in part support these beneficial effects; indeed, vitamin D treatment promote left ventricular reverse remodeling, as indicated by the reduction in ventricle volumes and improved function, and also improve survival in advanced CVD or CKD, but further randomized trials are expected to confirm whether supplementation of vitamin D may significantly influence the outcomes [77,78,79,80], apart from the difficulties already underlined in designing specific RCTs [81]. Although SSc patients selected for our study were already treated with a standard cholecalciferol 25.000 U per month, one-third out of them presented with vitamin D levels < 20 ng/mL. We found a significant bivariate correlation (Pearson’s) between CD34+ cell number and vitamin D levels; however, we cannot isolate the effects of vitamin D supplementation on CD34+ cells neither state whether the treatment could affect their number.

The study has several limitations. First of all, the relatively small sample size must be remarked; selected patients presented within a wide range of disease duration and they were on therapy with different strategies, following the clinical opportunity; however, we tried to select a homogeneous group of subjects, excluding smokers, patients with functional impairment or with comorbidities. This sample size limitation could affect the statistical analysis and does not allow us to perform sub-analyses; however, ours remains a well-stratified and rather homogeneous population. In this study, we compared some standard parameters and two novel potential biomarkers between SSc patients and healthy control subjects in an observational cross-sectional study; a sample size calculation was not previously performed. Overall, this could be considered a pilot study, and these preliminary results could not be immediately generalized to the population. Moreover, we focused on endocan and CD34+ cells to explore the vascular health in addition to sPAP estimation, but we did not evaluate other biomarkers (e.g., VEGF) potentially involved; furthermore, serum endocan levels were measured only in one time-point for this exploratory analysis. Further evaluations during the follow-up could be of help to correlate disease status, mechanical parameters, laboratory and novel biomarkers (CD34+ cells and endocan). Last, all selected SSc patients were already assuming a standard cholecalciferol therapy at enrolment; therefore, vitamin D supplementation could affect the results; in addition, while all selected patients were assuming ASA and nifedipine at standard dosage, three patients were prescribed bosentan and four patients sildenafil, and we were not able to take into consideration the potential effects of these therapies on our results.

Another aspect being considered is that right heart catheterization represents the gold standard for diagnosis and staging of PAH so far. However, non-invasive evaluation and biomarkers are also investigated to overcome the question of invasiveness.

Our study seems to enforce the findings on the potential role of endocan as a biomarker of vascular health in SSc; the relationships between endocan and other angiogenesis biomarkers should be evaluated by future prospective studies in order to investigate its ability in predicting vascular involvement, including PAH (possibly evaluated also by right heart catheterization), also in SSc.

## 5. Conclusions

Cardiovascular disease represents a prominent risk in autoimmune diseases, including systemic sclerosis. Standard echocardiography is currently used to estimate indirectly the involvement of pulmonary vessels and endothelium, while the gold standard for diagnosis and staging is the invasive measurement through the right heart catheterization. New markers of early cardiovascular and pulmonary artery involvement could be of help in assessing the overall health risk in this patient subset, also in the light of the high mortality due to this complication. Endocan and CD34+ progenitor cells might be suggested as potential additional markers of health, and disease status in immune disease, including systemic sclerosis.

## Figures and Tables

**Table 1:** Characteristic of the study population.

	SSc	Controls	*p*
	Mean ± SD	Mean ± SD	
Number	36	36	ns
Sex (f)	36	36	ns
BMI (kg/m^2^)	25.3 ± 4.43	24.9 ± 4.5	0.703
Age (years)	64.1 ± 12.5	62.3 ± 4.4	0.418
SBP (mmHg)	115 ± 14	120 ± 10	0.085
DBP (mmHg)	90 ± 15	80 ± 10	<0.001
Rodnan Skin Score	28.9 ± 10.2	-	-
Hb (g/dL)	12.5 ± 1.7	13.3 ± 0.8	0.013
Hs-CRP (mg/dL)	0.57 ± 0.95	0.30 ± 0.25	0.11
Creatinine (mg/dL)	0.77 ± 0.38	0.82 ± 0.09	0.444
Uric acid (mg/dL)	3.86 ± 1.05	2.9 ± 2.60	0.04
CD34+/μL (cells/mL)	2.68 ± 0.79	2.61 ± 0.17	0.604
Fibrinogen (mg/dL)	381.5 ± 100.1	276.9 ± 21.7	<0.001
VIT D μg/L (ng/mL)	27.34 ± 10.8	22.47 ± 2.6	0.010
Endocan (ng/mL)	365.6 ± 225.9	280.4 ± 68.7	0.034
EF (%)	59.9 ± 2.3	64.8 ± 0.99	0.001
E (cm/s)	72.9 ± 17.3	75.4 ± 7.3	0.427
A (cm/s)	71.85 ± 13.05	60.8 ± 5.9	<0.001
E/A (ratio)	1.04 ± 0.41	1.19 ± 0.34	0.101
E’ (cm/s)	9.66 ± 2.1	10.34 ± 1.62	0.128
S’ (cm/s)	8.5 ± 1.4	8.8 ± 2.0	0.464
E/E’ (ratio)	7.9 ± 2.31	7.17 ± 1.42	0.111
IVRT (msec)	86.1 ± 13.3	81.4 ± 10.7	0.103
RA volume (mL/m^2^)	24.2 ± 5.1	22.1 ± 5.0	0.082
RV wall thickness (mm)	2.2 ± 0.6	2.0 ± 0.3	0.078
RVOT prox diam (mm)	29.1 ± 2.8	26.3 ± 3	<0.001
TAPSE (mm)	15.7 ± 3.1	22.15 ± 4.3	<0.001
sPAP (mmHg)	34.1 ± 10.3	20.4 ± 1.3	<0.001

Values are mean ± SD. Abbreviations: BMI: body mass index; SBP: systolic blood pressure; DBP: diastolic blood pressure; Hs-CRP: high sensitivity C-reactive protein; ESR: erythrosedimentation rate; sPAP: pulmonary arterial pressure; EF: ejection fraction; E: early filling velocity on transmitral Doppler; A: late-atrial filling velocity on transmitral Doppler; E’: early diastolic myocardial velocity; S’: systolic myocardial velocity; IVRT: isovolumetric relaxation time; RA: right atrium; RV: right ventricle; RVOT: right ventricle outflow tract; TAPSE: tricuspid annular plane excursion; *p*: *p*-value level for two-tailed *t*-test; SSc patients versus controls.

**Table 2:** Clinical characteristics of patients with systemic sclerosis.

	SSc
Pattern of SSc, diffuse/limited (%)	24/12 (66.6/33.3%)
Disease duration, month	78.3 ± 61.9
Anticentromere antibodies (ACA), *n* (%)	16 (44.4%)
Anti-topoisomerase I (Anti-Scl70), *n* (%)	9 (25%)
Antinuclear positivity (ANA) *n* (%)	22 (61.1%)
Interstitial lung disease, *n* (%)	17 (47.2%)
Musculo-skeletal involvement, *n* (%)	19 (52.8%)
Renal crisis, *n* (%)	0
Esophageal reflux, *n* (%)	30 (83.3%)
Background therapy	36 (100%)
Aspirin 100 mg daily, *n* (%)	36 (100%)
Nifedipine 20 mg daily, *n* (%)	36 (100%)
Bosentan 62, 5/125 mg daily, *n* (%)	3 (8.33%)
Sildenafil 60 mg daily, *n* (%)	4 (11.1%)

**Table 3:** Linear regression model.

	Standardized Coefficients		Unstandardized Coefficients		
Model	B	SE	Beta	t	*p*
Fibrinogen	−0.005	0.001	−0.667	−4.732	0.000
Endocan	−0.002	0.001	−0.514	−3.171	0.004
sPAP	−0.035	0.012	−0.498	−2.980	0.006
SUA	−0.302	0.163	−0.383	−1.855	0.078
CRP	−0.080	0.158	−0.100	−0.504	0.619
Vitamin D	0.022	0.014	0.293	1.621	0.116

Dependent variable: CD34+ cell number.

**Table 4:** Multivariable regression model.

	Standardized Coefficients		Unstandardized Coefficients		
Model	B	SE	Beta	t	*p*
Costant	4.130	0.334		12.374	0.000
Endocan	−0.001	0.000	−412	−2.940	0.006
Fibrinogen	−0.003	0.001	−386	−2.753	0.009

Dependent variable: CD34+ cell number; excluded variables: sPAP.

**Table 5:** Linear regression model.

	Standardized Coefficients		Unstandardized Coefficients		
Model	B	SE	Beta	t	*p*
Endocan	0.033	0.007	0.607	4.519	0.000
Fibrinogen	0.054	0.017	0.471	3.161	0.003
CD34+ cells	−6.967	2.512	−0.424	−2.773	0.009
SUA	1.389	1.698	0.168	0.818	0.422
Rodnan SS	0.129	0.172	0.130	0.751	0.458
Vitamin D	0.007	0.163	0.007	0.041	0.967

Dependent variable: sPAP.

**Table 6:** Multivariable regression model.

	Standardized Coefficients		Unstandardized Coefficients		
Model	B	SE	Beta	t	*p*
Costant	18.463	3.278		5.632	0.000
Endocan	0.033	0.007	0.607	4.519	0.000

Dependent variable: sPAP; excuded variables: fibrinogen, CD34+ cells.

## Data Availability

All data generated or analyzed during the study are included in this published article.

## References

[1] Isola G., Williams R.C., Lo Gullo A., Ramaglia L., Matarese M., Iorio-Siciliano V., Cosio C., Matarese G. (2017). Risk association between scleroderma disease characteristics, periodontitis, and tooth loss. Clin. Rheumatol..

[2] Denton C.P., Khanna D. (2017). Systemic sclerosis. Lancet.

[3] Morrisroe K., Huq M., Stevens W., Rabusa C., Proudman S.M., Nikpour M., the Australian Scleroderma Interest Group (2016). Risk factors for development of pulmonary arterial hypertension in Australian systemic sclerosis patients: Results from a large multicenter cohort study. BMC Pulm. Med..

[4] Schermuly R.T., Ghofrani H.A., Wilkins M.R., Grimminger F. (2011). Mechanisms of disease: Pulmonary arterial hypertension. Nat. Rev. Cardiol..

[5] Guiducci S., Giacomelli R., Cerinic M.M. (2007). Vascular complications of scleroderma. Autoimmun. Rev..

[6] Hachulla E., Launay D., Boucly A., Mouthon L., de Groote P., Cottin V., Pugnet G., Prevot G., Bourlier D., Dauphin C. (2019). Survival Improved in Patients Aged ≤ 70 Years with Systemic Sclerosis-Associated Pulmonary Arterial Hypertension During the Period 2006 to 2017 in France. Chest.

[7] Colletti M., Galardi A., De Santis M., Guidelli G.M., Di Giannatale A., Di Luigi L., Antinozzi C. (2019). Exosomes in Systemic Sclerosis: Messengers Between Immune, Vascular and Fibrotic Components?. Int. J. Mol. Sci..

[8] Aragona C.O., Imbalzano E., Mamone F., Cairo V., Lo Gullo A., D’Ascola A., Sardo M.A., Scuruchi M., Basile G., Saitta A. (2016). Endothelial Progenitor Cells for Diagnosis and Prognosis in Cardiovascular Disease. Stem Cells Int..

[9] Rodriguez-Carrio J., Lopez P., Suarez A. (2018). Endothelial Progenitor Cells as Mediators of the Crosstalk between Vascular Repair and Immunity: Lessons from Systemic Autoimmune Diseases. Curr. Med. Chem..

[10] Spees J.L., Whitney M.J., Sullivan D.E., Lasky J.A., Laboy M., Ylostalo J., Prockop D.J. (2008). Bone marrow progenitor cells contribute to repair and remodeling of the lung and heart in a rat model of progressive pulmonary hypertension. FASEB J..

[11] Del Papa N., Pignataro F. (2018). The Role of Endothelial Progenitors in the Repair of Vascular Damage in Systemic Sclerosis. Front. Immunol..

[12] Rabquer B.J., Koch A.E. (2012). Angiogenesis and vasculopathy in systemic sclerosis: Evolving concepts. Curr. Rheumatol. Rep..

[13] Avouac J., Juin F., Wipff J., Couraud P.O., Chiocchia G., Kahan A., Boileau C., Uzan G., Allanore Y. (2008). Circulating endothelial progenitor cells in systemic sclerosis: Association with disease severity. Ann. Rheum. Dis..

[14] Andrigueti F.V., Arismendi M.I., Ebbing P.C., Kayser C. (2015). Decreased numbers of endothelial progenitor cells in patients in the early stages of systemic sclerosis. Microvasc. Res..

[15] Kuwana M., Okazaki Y., Yasuoka H., Kawakami Y., Ikeda Y. (2004). Defective vasculogenesis in systemic sclerosis. Lancet.

[16] Nevskaya T., Bykovskaia S., Lyssuk E., Shakhov I., Zaprjagaeva M., Mach E., Ananieva L., Guseva N., Nassonov E. (2008). Circulating endothelial progenitor cells in systemic sclerosis: Relation to impaired angiogenesis and cardiovascular manifestations. Clin. Exp. Rheumatol..

[17] Lo Gullo A., Aragona C.O., Scuruchi M., Versace A.G., Saitta A., Imbalzano E., Loddo S., Campo G.M., Mandraffino G. (2018). Endothelial progenitor cells and rheumatic disease modifying therapy. Vascul. Pharmacol..

[18] Lo Gullo A., Mandraffino G., Bagnato G., Aragona C.O., Imbalzano E., D’Ascola A., Rotondo F., Cinquegrani A., Mormina E., Saitta C. (2015). Vitamin D Status in Rheumatoid Arthritis: Inflammation, Arterial Stiffness and Circulating Progenitor Cell Number. PLoS ONE.

[19] Lo Gullo A., Mandraffino G., Imbalzano E., Mamone F., Aragona C.O., D’Ascola A., Loddo S., Cinquegrani A., Alibrandi A., Mormina E. (2014). Toll-like receptor 3 and interleukin 1beta expression in CD34+ cells from patients with rheumatoid arthritis: Association with inflammation and vascular involvement. Clin. Exp. Rheumatol..

[20] Lo Gullo A., Mandraffino G., Sardo M.A., D’Ascola A., Mamone F., Loddo S., Alibrandi A., Imbalzano E., Mandraffino R., Mormina E. (2014). Circulating progenitor cells in rheumatoid arthritis: Association with inflammation and oxidative stress. Scand. J. Rheumatol..

[21] Mok M.Y., Yiu K.H., Wong C.Y., Qiuwaxi J., Lai W.H., Wong W.S., Tse H.F., Lau C.S. (2010). Low circulating level of CD133+KDR+cells in patients with systemic sclerosis. Clin. Exp. Rheumatol..

[22] Del Papa N., Colombo G., Fracchiolla N., Moronetti L.M., Ingegnoli F., Maglione W., Comina D.P., Vitali C., Fantini F., Cortelezzi A. (2004). Circulating endothelial cells as a marker of ongoing vascular disease in systemic sclerosis. Arthritis Rheum..

[23] Allanore Y., Batteux F., Avouac J., Assous N., Weill B., Kahan A. (2007). Levels of circulating endothelial progenitor cells in systemic sclerosis. Clin. Exp. Rheumatol..

[24] Del Papa N., Quirici N., Scavullo C., Gianelli U., Corti L., Vitali C., Ferri C., Giuggioli D., Manfredi A., Maglione W. (2010). Antiendothelial cell antibodies induce apoptosis of bone marrow endothelial progenitors in systemic sclerosis. J. Rheumatol..

[25] Satoh K., Kagaya Y., Nakano M., Ito Y., Ohta J., Tada H., Karibe A., Minegishi N., Suzuki N., Yamamoto M. (2006). Important role of endogenous erythropoietin system in recruitment of endothelial progenitor cells in hypoxia-induced pulmonary hypertension in mice. Circulation.

[26] Wimalawansa S.J. (2016). Vitamin D and cardiovascular diseases: Causality. J. Steroid. Biochem. Mol. Biol..

[27] Jablonski K.L., Chonchol M., Pierce G.L., Walker A.E., Seals D.R. (2011). 25-Hydroxyvitamin D deficiency is associated with inflammation-linked vascular endothelial dysfunction in middle-aged and older adults. Hypertension.

[28] Grundmann M., Haidar M., Placzko S., Niendorf R., Darashchonak N., Hubel C.A., von Versen-Hoynck F. (2012). Vitamin D improves the angiogenic properties of endothelial progenitor cells. Am. J. Physiol. Cell Physiol..

[29] Cianciolo G., La Manna G., Cappuccilli M.L., Lanci N., Della Bella E., Cuna V., Dormi A., Todeschini P., Donati G., Alviano F. (2011). VDR expression on circulating endothelial progenitor cells in dialysis patients is modulated by 25(OH)D serum levels and calcitriol therapy. Blood Purif..

[30] Uberti F., Lattuada D., Morsanuto V., Nava U., Bolis G., Vacca G., Squarzanti D.F., Cisari C., Molinari C. (2014). Vitamin D protects human endothelial cells from oxidative stress through the autophagic and survival pathways. J. Clin. Endocrinol. Metab..

[31] Antico A., Tampoia M., Tozzoli R., Bizzaro N. (2012). Can supplementation with vitamin D reduce the risk or modify the course of autoimmune diseases? A systematic review of the literature. Autoimmun. Rev..

[32] Balta S., Mikhailidis D.P., Demirkol S., Ozturk C., Celik T., Iyisoy A. (2015). Endocan: A novel inflammatory indicator in cardiovascular disease?. Atherosclerosis.

[33] Oktar S.F., Guney I., Eren S.A., Oktar L., Kosar K., Buyukterzi Z., Alkan E., Biyik Z., Erdem S.S. (2019). Serum endocan levels, carotid intima-media thickness and microalbuminuria in patients with newly diagnosed hypertension. Clin. Exp. Hypertens..

[34] Sarrazin S., Adam E., Lyon M., Depontieu F., Motte V., Landolfi C., Lortat-Jacob H., Bechard D., Lassalle P., Delehedde M. (2006). Endocan or endothelial cell specific molecule-1 (ESM-1): A potential novel endothelial cell marker and a new target for cancer therapy. Biochim. Biophys. Acta.

[35] Lee H.G., Choi H.Y., Bae J.S. (2014). Endocan as a potential diagnostic or prognostic biomarker for chronic kidney disease. Kidney Int..

[36] Bechard D., Scherpereel A., Hammad H., Gentina T., Tsicopoulos A., Aumercier M., Pestel J., Dessaint J.P., Tonnel A.B., Lassalle P. (2001). Human endothelial-cell specific molecule-1 binds directly to the integrin CD11a/CD18 (LFA-1) and blocks binding to intercellular adhesion molecule-1. J. Immunol..

[37] Bessa J., Albino-Teixeira A., Reina-Couto M., Sousa T. (2020). Endocan: A novel biomarker for risk stratification, prognosis and therapeutic monitoring in human cardiovascular and renal diseases. Clin. Chim. Acta.

[38] Balta S., Mikhailidis D.P., Demirkol S., Ozturk C., Kurtoglu E., Demir M., Celik T., Turker T., Iyisoy A. (2014). Endocan—A novel inflammatory indicator in newly diagnosed patients with hypertension. Angiology.

[39] Icli A., Cure E., Cure M.C., Uslu A.U., Balta S., Mikhailidis D.P., Ozturk C., Arslan S., Sakiz D., Sahin M. (2016). Endocan Levels and Subclinical Atherosclerosis in Patients With Systemic Lupus Erythematosus. Angiology.

[40] Balta S., Demirkol S., Ozturk C., Yildirim A.O., Demir M., Celik T. (2016). The Relation Between Endocan Levels and Subclinic Atherosclerosis. Clin. Appl. Thromb. Hemost..

[41] Ying J., Zhou D., Gu T., Huang J. (2019). Endocan, a Risk Factor for Developing Acute Respiratory Distress Syndrome among Severe Pneumonia Patients. Can. Respir. J..

[42] Kechagia M., Papassotiriou I., Gourgoulianis K.I. (2016). Endocan and the respiratory system: A review. Int. J. Chron. Obstruct. Pulmon. Dis..

[43] Liang O.D., So E.Y., Egan P.C., Goldberg L.R., Aliotta J.M., Wu K.Q., Dubielecka P.M., Ventetuolo C.E., Reginato A.M., Quesenberry P.J. (2017). Endothelial to haematopoietic transition contributes to pulmonary arterial hypertension. Cardiovasc. Res..

[44] Van den Hoogen F., Khanna D., Fransen J., Johnson S.R., Baron M., Tyndall A., Matucci-Cerinic M., Naden R.P., Medsger T.A., Carreira P.E. (2013). 2013 classification criteria for systemic sclerosis: An American college of rheumatology/European league against rheumatism collaborative initiative. Ann. Rheum. Dis..

[45] Clements P., Lachenbruch P., Siebold J., White B., Weiner S., Martin R., Weinstein A., Weisman M., Mayes M., Collier D. (1995). Inter and intraobserver variability of total skin thickness score (modified Rodnan TSS) in systemic sclerosis. J. Rheumatol..

[46] Galie N., Humbert M., Vachiery J.L., Gibbs S., Lang I., Torbicki A., Simonneau G., Peacock A., Vonk Noordegraaf A., Beghetti M. (2015). 2015 ESC/ERS Guidelines for the diagnosis and treatment of pulmonary hypertension: The Joint Task Force for the Diagnosis and Treatment of Pulmonary Hypertension of the European Society of Cardiology (ESC) and the European Respiratory Society (ERS): Endorsed by: Association for European Paediatric and Congenital Cardiology (AEPC), International Society for Heart and Lung Transplantation (ISHLT). Eur. Respir. J..

[47] Khanna D., Furst D.E., Clements P.J., Allanore Y., Baron M., Czirjak L., Distler O., Foeldvari I., Kuwana M., Matucci-Cerinic M. (2017). Standardization of the modified Rodnan skin score for use in clinical trials of systemic sclerosis. J. Scleroderma Relat. Disord..

[48] Lang R.M., Badano L.P., Mor-Avi V., Afilalo J., Armstrong A., Ernande L., Flachskampf F.A., Foster E., Goldstein S.A., Kuznetsova T. (2015). Recommendations for cardiac chamber quantification by echocardiography in adults: An update from the American Society of Echocardiography and the European Association of Cardiovascular Imaging. Eur. Hearth J. Cardiovasc. Imaging.

[49] Pugnet G., Marjanovic Z., Deligny C., Boussardon K., Benzidia I., Puyade M., Lansiaux P., Vandecasteele E., Smith V., Farge D. (2018). Reproducibility and Utility of the 6-minute Walk Test in Systemic Sclerosis. J. Rheumatol..

[50] Nagel C., Marra A.M., Benjamin N., Blank N., Cittadini A., Coghlan G., Distler O., Denton C.P., Egenlauf B., Fiehn C. (2019). Reduced Right Ventricular Output Reserve in Patients With Systemic Sclerosis and Mildly Elevated Pulmonary Artery Pressure. Arthritis Rheumatol..

[51] Mandraffino G., Imbalzano E., Sardo M.A., D’Ascola A., Mamone F., Lo Gullo A., Alibrandi A., Loddo S., Mormina E., David A. (2014). Circulating progenitor cells in hypertensive patients with different degrees of cardiovascular involvement. J. Hum. Hypertens..

[52] Holick M.F., Binkley N.C., Bischoff-Ferrari H.A., Gordon C.M., Hanley D.A., Heaney R.P., Murad M.H., Weaver C.M. (2011). Evaluation, treatment, and prevention of vitamin D deficiency: An Endocrine Society clinical practice guideline. J. Clin. Endocrinol. Metab..

[53] Yilmaz M.I., Siriopol D., Saglam M., Kurt Y.G., Unal H.U., Eyileten T., Gok M., Cetinkaya H., Oguz Y., Sari S. (2014). Plasma endocan levels associate with inflammation, vascular abnormalities, cardiovascular events, and survival in chronic kidney disease. Kidney Int..

[54] Balta I., Balta S., Demirkol S., Mikhailidis D.P., Celik T., Akhan M., Kurt O., Kurt Y.G., Aydin I., Kilic S. (2013). Elevated serum levels of endocan in patients with psoriasis vulgaris: Correlations with cardiovascular risk and activity of disease. Br. J. Dermatol..

[55] Balta I., Balta S., Koryurek O.M., Demirkol S., Mikhailidis D.P., Celik T., Cakar M., Kucuk U., Eksioglu M., Kurt Y.G. (2014). Serum endocan levels as a marker of disease activity in patients with Behcet disease. J. Am. Acad. Dermatol..

[56] Balanescu P., Ladaru A., Balanescu E., Voiosu T., Baicus C., Dan G.A. (2016). Endocan, Novel Potential Biomarker for Systemic Sclerosis: Results of a Pilot Study. J. Clin. Lab. Anal..

[57] Zhao H., Xue Y., Guo Y., Sun Y., Liu D., Wang X. (2017). Inhibition of endocan attenuates monocrotaline-induced connective tissue disease related pulmonary arterial hypertension. Int. Immunopharmacol..

[58] Fujita M., Shannon J.M., Irvin C.G., Fagan K.A., Cool C., Augustin A., Mason R.J. (2001). Overexpression of tumor necrosis factor-alpha produces an increase in lung volumes and pulmonary hypertension. Am. J. Physiol. Lung Cell Mol. Physiol..

[59] Tanaka K., Sata M. (2009). Role of vascular progenitor cells in cardiovascular disease. Curr. Pharm. Des..

[60] Westerweel P.E., Verhaar M.C. (2009). Endothelial progenitor cell dysfunction in rheumatic disease. Nat. Rev. Rheumatol..

[61] Del Papa N., Quirici N., Soligo D., Scavullo C., Cortiana M., Borsotti C., Maglione W., Comina D.P., Vitali C., Fraticelli P. (2006). Bone marrow endothelial progenitors are defective in systemic sclerosis. Arthritis Rheum..

[62] Diller G.P., van Eijl S., Okonko D.O., Howard L.S., Ali O., Thum T., Wort S.J., Bedard E., Gibbs J.S., Bauersachs J. (2008). Circulating endothelial progenitor cells in patients with Eisenmenger syndrome and idiopathic pulmonary arterial hypertension. Circulation.

[63] Schiavon M., Fadini G.P., Lunardi F., Agostini C., Boscaro E., Calabrese F., Marulli G., Rea F. (2012). Increased tissue endothelial progenitor cells in end-stage lung diseases with pulmonary hypertension. J. Hearth Lung Transpl..

[64] Ormiston M.L., Deng Y., Stewart D.J., Courtman D.W. (2010). Innate immunity in the therapeutic actions of endothelial progenitor cells in pulmonary hypertension. Am. J. Respir. Cell Mol. Biol..

[65] Conese M., Carbone A., Castellani S., Di Gioia S. (2013). Paracrine effects and heterogeneity of marrow-derived stem/progenitor cells: Relevance for the treatment of respiratory diseases. Cells Tissues Organs.

[66] Xia L., Fu G.S., Yang J.X., Zhang F.R., Wang X.X. (2009). Endothelial progenitor cells may inhibit apoptosis of pulmonary microvascular endothelial cells: New insights into cell therapy for pulmonary arterial hypertension. Cytotherapy.

[67] Smadja D.M., Mauge L., Gaussem P., d’Audigier C., Israel-Biet D., Celermajer D.S., Bonnet D., Levy M. (2011). Treprostinil increases the number and angiogenic potential of endothelial progenitor cells in children with pulmonary hypertension. Angiogenesis.

[68] Li Y.C., Kong J., Wei M., Chen Z.F., Liu S.Q., Cao L.P. (2002). 1,25-Dihydroxyvitamin D(3) is a negative endocrine regulator of the renin-angiotensin system. J. Clin. Investig..

[69] Lai S., Coppola B., Dimko M., Galani A., Innico G., Frassetti N., Mariotti A. (2014). Vitamin D deficiency, insulin resistance, and ventricular hypertrophy in the early stages of chronic kidney disease. Ren. Fail.

[70] Covic A., Voroneanu L., Goldsmith D. (2010). The effects of vitamin D therapy on left ventricular structure and function—Are these the underlying explanations for improved CKD patient survival?. Nephron Clin. Pract..

[71] Gardner D.G., Chen S., Glenn D.J. (2013). Vitamin D and the heart. Am. J. Physiol. Regul. Integr Comp. Physiol..

[72] Bickford P.C., Tan J., Shytle R.D., Sanberg C.D., El-Badri N., Sanberg P.R. (2006). Nutraceuticals synergistically promote proliferation of human stem cells. Stem Cells Dev..

[73] Schroder-Heurich B., von Hardenberg S., Brodowski L., Kipke B., Meyer N., Borns K., von Kaisenberg C.S., Brinkmann H., Claus P., von Versen-Hoynck F. (2019). Vitamin D improves endothelial barrier integrity and counteracts inflammatory effects on endothelial progenitor cells. FASEB J..

[74] Hammer Y., Soudry A., Levi A., Talmor-Barkan Y., Leshem-Lev D., Singer J., Kornowski R., Lev E.I. (2017). Effect of vitamin D on endothelial progenitor cells function. PLoS ONE.

[75] Wara A.K., Foo S., Croce K., Sun X., Icli B., Tesmenitsky Y., Esen F., Lee J.S., Subramaniam M., Spelsberg T.C. (2011). TGF-beta1 signaling and Kruppel-like factor 10 regulate bone marrow-derived proangiogenic cell differentiation, function, and neovascularization. Blood.

[76] Latic N., Erben R.G. (2020). Vitamin D and Cardiovascular Disease, with Emphasis on Hypertension, Atherosclerosis, and Heart Failure. Int. J. Mol. Sci..

[77] Witte K.K., Byrom R., Gierula J., Paton M.F., Jamil H.A., Lowry J.E., Gillott R.G., Barnes S.A., Chumun H., Kearney L.C. (2016). Effects of Vitamin D on Cardiac Function in Patients With Chronic HF: The VINDICATE Study. J. Am. Coll. Cardiol..

[78] Alfieri C., Vettoretti S., Ruzhytska O., Gandolfo M.T., Cresseri D., Campise M., Caldiroli L., Favi E., Binda V., Messa P. (2020). Vitamin D and subclinical cardiac damage in a cohort of kidney transplanted patients: A retrospective observational study. Sci. Rep..

[79] Czaya B., Seeherunvong W., Singh S., Yanucil C., Ruiz P., Quiroz Y., Grabner A., Katsoufis C., Swaminathan S., Abitbol C. (2019). Cardioprotective Effects of Paricalcitol Alone and in Combination With FGF23 Receptor Inhibition in Chronic Renal Failure: Experimental and Clinical Studies. Am. J. Hypertens..

[80] Gluba-Brzózka A., Franczyk B., Ciałkowska-Rysz A., Olszewski R., Rysz J. (2018). Impact of Vitamin D on the Cardiovascular System in Advanced Chronic Kidney Disease (CKD) and Dialysis Patients. Nutrients.

[81] Boucher B.J., Grant W.B. (2020). Difficulties in designing randomised controlled trials of vitamin D supplementation for reducing acute cardiovascular events and in the analysis of their outcomes. Int. J. Cardiol. Hearth Vasc..

